# Supplementary approaches to perinatal depression: a review of pathogenesis, herbal interventions, and dietary supplements

**DOI:** 10.3389/fpsyg.2025.1529339

**Published:** 2025-05-21

**Authors:** Quancheng Yang, Yi Lv, Shenrong Gao, Yu Zhang, Xuejia Zhai

**Affiliations:** ^1^Department of Pharmacy, Union Hospital, Tongji Medical College, Huazhong University of Science and Technology, Wuhan, China; ^2^Hubei Province Clinical Research Center for Precision Medicine for Critical Illness, Wuhan, China; ^3^Department of Pharmacy, Hubei Hospital of Traditional Chinese Medicine, Wuhan, China

**Keywords:** perinatal depression, pathophysiological mechanisms, non-drug intervention, herbal remedies, dietary supplements

## Abstract

Although perinatal depression (PND) has garnered increasing attention, few specific pharmacological treatments exist, particularly for breastfeeding mothers concerned about antidepressant safety. The misconception that “natural is harmless” merits caution; herbal remedies and dietary supplements should be regarded as supplementary interventions pending robust safety evidence. This review summarizes recent advances in PND pathogenesis (neurotransmitter dysregulation, inflammation, hormonal imbalance, and microbiota alterations) and emerging drug development strategies, alongside clinical evidence for herbal and dietary supplements. Randomized controlled trial (RCT) findings reveal that while interventions like saffron and vitamin D show promise, significant limitations persist, including inconsistent efficacy, limited long-term safety data, and potential interactions with perinatal physiology. Caution is warranted until comprehensive studies validate the safety and reliability of natural interventions. This review underscores the need for rigorous trials to identify safe, effective PND treatments, particularly for vulnerable populations.

## 1 Introduction

Perinatal psychiatric disorders encompass a spectrum of mental health challenges occurring during pregnancy and postpartum, varying in clinical presentation and severity. Among these, baby blues (postpartum blues) represent the most prevalent yet transient condition, affecting nearly 40% of women within the first week postpartum (Rezaie-Keikhaie et al., 2020). Characterized by mood lability, anxiety, and irritability, baby blues typically resolve spontaneously without intervention. In contrast, postnatal psychosis, a severe neuropsychiatric disorder, involves hallucinations, delusions, and manic episodes, necessitating immediate psychiatric care (Perry et al., 2021). Perinatal depression (also known as postpartum depression) is one of the most common obstetric complications for mothers during pregnancy and after delivery. According to a study by Al-Abri et al. (2023), which analyzed 128 systematic reviews, the overall average prevalence of perinatal depression is 26.3% (SD = 11.6, median = 23.8%). This indicates that one in four perinatal women experiences depression. The burden of PND is particularly pronounced among high-risk populations, including those exposed to intimate partner violence, HIV infection, or the aftermath of natural disasters (Roddy Mitchell et al., 2023). Beyond its immediate psychological burden, untreated PND disrupts maternal–infant bonding, impairs child cognitive development, and increases long-term risks of chronic mood disorders. Despite its profound public health implications, PND universally remains underdiagnosed and undertreated, with fewer than 20% of affected women receiving adequate care, especially in low-resource settings where care accessibility is severely limited (Roddy Mitchell et al., 2023; Payne and Maguire, 2019). Studies have indicated that the causes of PND can have various sources, including physiological ones, such as hormone fluctuations (Worthen and Beurel, 2022), psychological ones, such as family pressures and a lack of social support (Upadhyay et al., 2017; Pillai et al., 2023), such as societal expectations (Klainin and Arthur, 2009), and genetic factors, such as those involved in monoamine neurotransmitters, key molecules of the HPA axis and kynurenine pathways, as well as genes associated with immune regulation (Mehta et al., 2021). Unfortunately, existing treatments lack specificity for PND (Frieder et al., 2019). Despite the extensive understanding of these factors, current treatment options remain limited and often inadequate, with antidepressants being the primary pharmaceutical approach. However, concerns over potential risks to both maternal health and infant safety—especially during breastfeeding—have led many women to seek alternative therapies (Dubreucq et al., 2022; Johansen et al., 2019).

First-line pharmacological interventions for PND—primarily selective serotonin reuptake inhibitors (SSRIs) such as sertraline and fluoxetine—are largely extrapolated from major depressive disorder (MDD) protocols (Brown et al., 2021). While SSRIs demonstrate moderate efficacy, their use in perinatal populations is complicated by two critical factors: Firstly, up to 5–10% of maternal SSRI doses transfer to breast milk, raising concerns about neonatal neurobehavioral outcomes, albeit with conflicting evidence (Heinonen et al., 2021). Secondly, most women with PND face the dilemma of choosing between the risk of depression and the risks of antidepressants. While risks of antidepressants are often overstated, the consequences of untreated depression during pregnancy and postpartum are gravely underestimated. Untreated moderate-to-severe perinatal depression poses critical threats to maternal and infant wellbeing, including heightened risks of maternal substance use and suicidal ideation (Latendresse et al., 2017; Stohl et al., 2016). The impact on the long-term development of newborns could make sick mothers more careful about the choice of drug treatment. Thus, being able to treat PND while breastfeeding is an important challenge at present. This therapeutic impasse has fueled interest in non-pharmacological alternatives, particularly plant-based remedies (e.g., St. John’s wort, saffron) and dietary supplements (e.g., omega-3 fatty acids, vitamin D) because they tend to be perceived as less intrusive and “safer.” However, this perception is fraught with scientific ambiguity and caution should always be exercised until sufficient reliable studies.

In particular, although herbal medicines (including Chinese herbs) are often considered “natural remedies, “their pharmacological components can still be transmitted through breast milk or cause adverse reactions. Therefore, this article will carefully distinguish the potential risks of plant-derived therapy as a “drug” when discussing it, and emphasize the need for caution in its clinical application. The use of herbal medicine in treating mental disorders has a long tradition. St. John’s wort (SJW), a traditional European herbal medicine remedy, has been widely used to treat anxiety, depression, and even sleep disorders (Canenguez Benitez et al., 2022). Its low level of excretion into breast milk makes it a popular choice among postpartum mothers (Avila et al., 2018). However, the fact that part of the use of SJW exceeds prescribed dosages and lacks guidance from medical professionals creates safety risks. Saffron is commonly used by women in the field of food and medicine; it is taken mainly from the flower’s stigma. Clinical studies predominantly focus on its anti-inflammatory, antioxidant, antidepressant, and anti-anxiety activities (El Midaoui et al., 2022). In recent years, studies have evaluated its safety and compared its efficacy with SSRIs in the treatment of PND (Tabeshpour et al., 2017; Kashani et al., 2017). Additionally, herbs like lavender and basil, known for their volatile essential oils, are often used in aromatherapy to alleviate PND (Cho and Kim, 2023). Despite their popularity, these “natural” remedies are not without risks. SJW, for instance, can interact with other medications, reducing their efficacy, and many herbal preparations show significant variability in their bioactive compounds.

Although mothers in many cultures follow special care routines during the perinatal period, including less physical activity and changes in diet, recent evidence suggests that changes in diet are beneficial. Two recent cross-sectional studies from China with 939 and 1,659 participants, respectively, collected data on 24-h meal recall and food frequency to assess the association between dietary patterns and PND risk (Yang et al., 2021; Cao et al., 2020). The results showed that diet imbalance was associated with an increased risk of PND, while the healthy pattern of vegetables, fruits, and nuts was significantly associated with a decreased risk of PND; this was similar to the conclusion of a recent cross-sectional study in Iran (Dehghan-Banadaki et al., 2023). Therefore, diet during the perinatal period and the dietary supplements chosen are critically important.

This review addresses three critical gaps: (1) synthesizing recent advances in PND pathogenesis (e.g., neurosteroid fluctuations, gut-brain axis dysregulation) to establish mechanistic rationales for alternative therapies; (2) critically evaluating the safety profiles of herbal and dietary interventions, with emphasis on lactation risks and herb-drug interactions; and (3) proposing a clinically actionable framework to prioritize evidence-backed interventions (e.g., saffron) while flagging those requiring safety validation (e.g., TCM). By bridging preclinical insights and clinical realities, this work aims to guide clinicians and patients in navigating the complex trade-offs between conventional and alternative PND treatments.

## 2 Literature review

We searched PubMed, Web of Science, and Cochrane Library from 2000 to 2024 using the following key search terms: “postpartum depression,” “perinatal depression,” “treatment efficacy,” “pharmacological interventions,” “psychological therapies,” “alternative medicine,” “herbal medicine,” “psychotherapy approaches,” “maternal mental health,” “breastfeeding safety,” “randomized controlled trials,” “meta-analysis,” “longitudinal studies,” and “observational research.” We prioritized randomized controlled trials (RCTs) and meta-analyses, followed by high-quality longitudinal and cohort studies. We included all English language, peer-reviewed publications with sufficient methodological rigor, applying stricter thresholds for study size and quality in pharmacological intervention studies compared to psychological therapies. When multiple large RCTs were available, we did not include smaller case series or uncontrolled studies. We incorporated pilot studies and observational research only when robust clinical trials were lacking. We also performed manual reference checking of all included articles to identify additional relevant studies.

### 2.1 Prenatal depression pathogenesis

In the Diagnostic and Statistical Manual of Mental Disorders (DSM-5) and the International Classification of Diseases (ICD-10), PND is considered a specific period of depression; that is, symptoms occur within 4 weeks after giving birth or during pregnancy. Therefore, although some evidence supports PND as a unique disorder (Di Florio and Meltzer-Brody, 2015), it still shares the same diagnostic criteria as other non-perinatal depression, such as anxiety, stress, and emotional impairment, among others. Multiple cohort studies have shown that the increased risk of PND is independently related to psychosocial, genetic, and perinatal complications (Koutra et al., 2014; Koutra et al., 2018; Walker et al., 2021). The studies on this have looked at the mental fragility and emotional damage caused by PND, and explored the physiological material changes and functional impairment of pregnant mothers (Stewart and Vigod, 2019).

#### 2.1.1 The neurotransmitter

##### 2.1.1.1 Serotonin (5-HT)

In As an affective disorder, PND is characterized by low mood. It is widely believed that emotional changes are closely related to monoamine neurotransmitters. A number of clinical and pre-clinical studies have focused on the correlation between low 5-HT levels and depressive emotions (Jenkins et al., 2016; Young and Leyton, 2002; Toker et al., 2010). At the same time, the transmission of monoamine neurotransmitters, represented by 5-HT, is considered to be highly correlated with the onset and development of PND. Abnormal serotonin neurotransmission is viewed as an important factor in the onset of depression, so SSRIs, targeting 5-HT transporters, are widely used as first-line drugs for antidepression treatment (Latendresse et al., 2017; Haase and Brown, 2015; Reilly et al., 1997). Studies have shown that pregnant and postpartum women have high levels of serotonin and its metabolites in cerebrospinal fluid, and in late pregnancy and early postnatal, this is accompanied by low levels of tryptophan (Pawluski et al., 2019). In a longitudinal cohort study, Borgsted et al. (2022) evaluated the effect of the central serotonin system on maternal mental distress during the prenatal to postnatal transition. Results of high-performance liquid-chromatography (HPLC) analysis of maternal cerebrospinal fluid showed that higher levels of postpartum mental distress were positively correlated, at trend level, with levels of serotonin metabolite 5-hydroxy indole acetic acid (5-HIAA) in high-yield pre-cerebrospinal fluid (Borgsted et al., 2022).

##### 2.1.1.2 Dopamine

During pregnancy and motherhood, there are dynamic, continuous changes in brain structure, function, and behavior. This includes the adaptation of the brain’s reward system, the mesolimbic dopamine (DA) system. These alterations are thought to be adaptive and promote the initiation and maintenance of maternal behavior, which is essential for offspring survival (Barba-Muller et al., 2019). A number of perinatal studies on rodents have focused on time-dependent changes in DA function, showing that increasing perinatal dopaminergic activity is crucial for maternal and infant care (Stolzenberg and Numan, 2011; Rincon-Cortes and Grace, 2020). In other words, PND can occur if this adaptive change is disrupted or defective. In their study, Post and Leuner (2019) summarized nine neuroimaging studies on the mesolimbic dopamine system in patients with PND. Their results showed that the striatum with high depressive symptoms had weakened function and was more sluggish in responding to positive stimuli, which was consistent with the anhedonia symptom of PND (Post and Leuner, 2019).

##### 2.1.1.3 GABA

Some scientists have been inspired to understand depression and develop therapeutic drugs from non-monoamine ideas. In this process, excitatory/inhibitory (E/I) regulation based on non-monoamine targets γ-aminobutyric (GABA) acid and glutamatergic (Glu) has become an important non-monoamine drug development strategy (Li, 2020). Based on studies on rodents, researchers have shown that the structure and function of GABA_A_ receptors in the brain undergo significant changes in response to the fluctuation of neuroactive steroid levels during the perinatal period (Sanna et al., 2009). Some speculate that neurological and psychiatric disorders during this period may relate to the failure of these receptors to adapt to the changes (Maguire et al., 2009). Notably, Maguire and Mody (2008) identified a GABA_A_ receptor subunit-deficient mouse model in their study. The mice exhibited symptoms similar to depression and anxiety only after delivery, suggesting that this could become a specific model for PND (rather than major depression) (Maguire and Mody, 2008). Neuroactive steroids (NAS) are modulators of the GABA system. The neuroactive steroid, allopregnanolone, has been shown to be involved in emotional regulation and play an important role in the pathophysiology of mood disorders, such as depression or anxiety (Meltzer-Brody and Kanes, 2020). Deligiannidis et al. (2016) evaluated plasma NAS and GABA levels in perinatal women at risk for PND in a prospective study. Their results show that perinatal GABA concentrations were lower in AR-PND women compared with the healthy cohorts and negatively correlated with the Hamilton Depression Rating Scale (HAM-D17) and the Hamilton Anxiety Rating Scale (HAM-A) scores. In their study, however, no correlation was found between individual NAS levels and GABA concentration. The GABA level during the whole perinatal period was also not monitored, so it was difficult to further reveal the potential dynamic change process of perinatal GABA level (Deligiannidis et al., 2016).

#### 2.1.2 Nerve inflammation

Pregnancy is closely related to a body’s inflammatory response, and implanting and delivery are typical inflammatory processes (Romero et al., 2007). During labor, the uterus shifts from a relatively stable state to a relatively active state from the accumulation of a large number of proinflammatory signals (Leimert et al., 2021). Normal pregnancy relies on a rigorous balance of proinflammatory and anti-inflammatory signals, and abnormal inflammatory signals can lead to severe obstetric complications, such as preterm birth and eclampsia (Kalagiri et al., 2016; Challis et al., 2009). Given the high levels of proinflammatory cytokines in pregnant women during the perinatal trimester, they are also at a high risk for sleep disorders, postpartum pain, and even PND (Kendall-Tackett, 2007). In recent studies, emerging evidence has suggested that PND is also an inflammatory disease (Osborne and Monk, 2013).

Sluiter et al. (2020) analyzed levels of inflammatory epigenetic markers in blood samples from 148 Latina women in the United States. They found that high levels of DNA methylation in the FOXP3 and TNF-α promoter regions correlated prenatal perceptual discrimination with PND and anxiety symptoms (Sluiter et al., 2020). For the first time, Boufidou et al. (2009) evaluated elevated levels of the pro-inflammatory cytokines IL-6 and TNF-α in maternal cerebrospinal fluid (CSF). The results revealed a partial potential association between brain inflammation and depressive symptoms, given the high sensitivity of CSF tests to the central nervous system. Notably, Sathyanarayanan et al. (2019) studied inflammation levels in women with their first episode of PND. Their results showed that IL-6 levels rose immediately in healthy women (HP group) and in depressed women (PP group), likely representing a normal perinatal physiological response. However, the increase of IL-8 in the PP and HP groups was not expected compared with healthy non-maternal (HNP) groups. Thus, this may partially reveal changes in the specific inflammatory levels of perinatal depression (Sathyanarayanan et al., 2019). Notably, mothers with a history of MDD are accompanied by sensitization of the inflammatory response system (IRS), and the phenomenon of immune activation is more severe after delivery (Maes et al., 2001).

#### 2.1.3 Hormone levels

As the body supports fetal development and prepares for maternal delivery, women undergo significant neuroendocrine changes during the perinatal period. Specifically, we look at the changes in gonadal hormones and glucocorticoids. Progesterone, isoprogesterone, and estradiol remain high for a long time during pregnancy, but decline rapidly during delivery (Galea and Frokjaer, 2019). Meanwhile, the plasma glucocorticoid levels of women during pregnancy are more than two times higher than those of non-pregnant women (Lindsay and Nieman, 2005). Although these changes are preparing women for motherhood, studies have shown that pregnant women are more vulnerable and susceptible to hormonal fluctuations, and that the endocrine changes associated with childbirth play a direct role in PND in certain women (Galea and Frokjaer, 2019).

#### 2.1.4 Sex hormone

Perinatal estrogen decline leads to a state of estrogen withdrawal, which may be responsible for the emotional and behavioral changes in perinatal mothers. The estrogen withdrawal hypothesis is one of the most common explanations for the pathogenesis of PND (Schmidt et al., 2015). Many studies of rodents have shown that perinatal estrogen withdrawal (estradiol) leads to symptoms of depression and anxiety after delivery, and that sustained high levels of estradiol treatment can reverse these depression-like behaviors (Stoffel and Craft, 2004; Galea et al., 2001). To identify early biomarkers of PND in a longitudinal discovery cohort, Mehta et al. (2014) conducted a genome-wide study of 62 psychopathological women. Researchers identified 116 transcripts with differential expression, predicting PPD with 88% accuracy in both discovery and replication cohorts. These transcriptomes were significantly enriched in estrogen signaling pathways (represented by SP1 and TAF6). In addition, the estrogen receptor ESR1 site is the only transcription factor binding site that is significantly enriched. At the same time, PND women showed increased sensitivity to estrogen signals. It is worth mentioning that plasma estradiol and estriol levels of women in different groups did not show differences (Mehta et al., 2014).

#### 2.1.5 Glucocorticoid

One of the most credible findings in depression-related psychiatry over the years has been the dysregulation of the hypothalamic–pituitary–adrenal axis (HPA) in major depression (Alam et al., 2024). Although PND is generally considered a subtype of major depression, the HPA disorder in PND is significantly different from that in MDD (Glynn et al., 2013). The data show that the changes in the female endocrine stress system during pregnancy mainly come from the production and growth of a new organ, the placenta. Different from the negative feedback effect of glucocorticoids on the hypothalamus, the placenta secretes the additional placental corticotropin-releasing hormone (CRH) during pregnancy, and glucocorticoids activate the placenta and stimulate CRH synthesis (Frim et al., 1988). This positive feedback loop leads to significant increases in the adrenocorticotropic hormone (ACTH), cortisol, and the corticotropin-releasing hormone in the mother throughout pregnancy (Lindsay and Nieman, 2005). Animal models of postpartum depression studying the HPA axis have focused on pregnancy stress and/or exposure to high levels of corticosterone. Chronic mild stress has been found to induce elevated corticosterone and estrogen levels at the end of pregnancy, and lead to postpartum depressive behavior in these mice (Brummelte and Galea, 2010a; Brummelte and Galea, 2010b).

#### 2.1.6 Gut microbiota

The gut-brain axis is a bidirectional connection between the brain and the gastrointestinal tract. There is growing evidence that gut microbiota is involved in a number of mental disorders, including depression (Alam et al., 2024) Gut microbiota acts on the central nervous system (CNS) in three main ways: 1. The microbiome directly stimulates neurons to transmit signals to the brain via the vagus nerve; 2. The gut microbiota influence the regulation of neurotransmitters and glucocorticoids (HPA axis) through neuroendocrine pathways (Marano et al., 2023); 3. The gut microbiota affect the blood–brain barrier (BBB) and CNS function through immune cells. A prospective cohort study of 90 pregnant women found that microbial diversity was significantly associated with greater intestinal symptoms and feelings of helplessness in the late perinatal period (Long et al., 2023). Multiple studies evaluating the gut microbiota of patients with PND versus healthy women have shown significant differences between the two groups. Chen et al. (2021) analyzed stool samples from depressed women and healthy subjects. Bacteroidetes, Proteobacteria, and Clostriobacteria were highly enriched in patients with MDD, while firmicutes and actinobacteria were consistently higher in the control group (Chen et al., 2021). The number of microbiome specific genera in the stool of patients with PND correlated with clinical indicators and sex hormone levels (Zhou et al., 2020). However, because of limitations of sample size, homogeneity, age, diet, and other factors, the conclusions of these studies on microbial diversity changes in PPD patients are still controversial. More general consensus needs to be obtained in more reliable large-scale clinical trials.

### 2.2 Drug therapy for PND

In terms of current PND interventions, most are derived from the treatment of MDD (represented by SSRIs), with selective serotonin reuptake inhibitors (SSRIs) like sertraline and escitalopram serving as first-line therapies due to their established efficacy in reducing depressive symptoms via serotonin reuptake inhibition (O’Hara et al., 2019; Milgrom et al., 2015; Wisner et al., 2004; Misri et al., 2012). Few drugs are specifically approved for perinatal depression. Emerging therapies, as summarized in Table 1, include neuroactive steroids (e.g., brexanolone) showing symptom reduction in Phase 3 trials (Meltzer-Brody et al., 2018; Kanes et al., 2017; Deligiannidis et al., 2021). Hormonal interventions like transdermal estradiol with mixed evidence for efficacy (Li et al., 2020; Wisner et al., 2015; Gregoire et al., 1996). For women who are breastfeeding, antidepressants used for PND must be careful of adverse reactions during lactation. Future directions must prioritize personalized pharmacogenomic strategies and rigorous safety profiling in perinatal populations. The clinical studies (RCT dominated) on the drug treatment or prevention of PND to date are presented in Table 1.

**Table 1:** Clinical trials of drug treatment of perinatal depression.

Drug	Scale	Clinical trial phase	ClinicalTrials.gov identifier	References
Brexanolone injection	HAM-D	Phase 3 trials	NCT02942004 (study 1) NCT02942017 (study 2)	Meltzer-Brody et al. (2018)
Brexanolone (USAN; formerly SAGE-547 injection)	HAM-D	Phase 2 trials	NCT02614547	Kanes et al. (2017)
Zuranolone (SAGE-217)	HAMD-17	Phase 3 trials	NCT02978326	Deligiannidis et al. (2021)
Transdermal estradiol	BDI, EPDS, HAM-D	Placebo controlled treatment trial		Li et al. (2020)
Oxytocin	EPDS, SCID-5-CV	RCT	RBR-3TV5MR	Donadon et al. (2021)
Transdermal estradiol	SIGH-ADS29	RCT		Wisner et al. (2015)
Transdermal estrogen	EPDS, SADS	RCT		Gregoire et al. (1996)
Sertraline	HAMD-17	RCT	NCT00602355	O’Hara et al. (2019)
Sertraline	EPDS	RCT	NCT02122393	Milgrom et al. (2015)
Sertraline	HAM-D	RCT		Wisner et al. (2004)
Sertraline Sertraline add-on to psychotherapy	HAM-D, CGI MADRS	RCT RCT	NCT01028482	Hantsoo et al. (2014) and Bloch et al. (2012)
Nortriptyline versus sertraline	HRDS	RCT		Wisner et al. (2006)
Escitalopram	MADRS	Open-Label Study		Misri et al. (2012)
Paroxetine	CGI	RCT		Yonkers et al. (2008)
Ketamine	EPDS	RCT	ChiCTR-ROC-17,012,944	Ma et al. (2019)
Bupropion SR	HAMD, CGI	open-label trial		Nonacs et al. (2005)
Dexmedetomidine	EPDS	RCT		Yu et al. (2019)

HAM-D, Hamilton Depression Rating Scale; HAMD-17, Hamilton Depression Rating Scale; EPDS, Edinburgh Postnatal Depression Scale; STAI, State–trait anxiety inventory; DHA, Docosahexaenoic acid; BSID, Bayley Scales of Infant development; HDRS, Hamilton Depression Rating Scale; PSQS, Postpartum Sleep Quality Scale; BDI-II, Beck Depression Inventory-Second Edition; BAI, Beck Anxiety Inventory; SCID-5-CV, Structured Clinical Interview for DSM-5 Disorders; SIGH-ADS29, Hamilton Depression Rating Scale—Atypical Depression Symptoms Version; MADRS, Montgomery-Asberg Depression Rating Scale.

### 2.3 Botanicals/natural products

Given the unique pathophysiological mechanisms of postpartum depression and the special physiological conditions of perinatal women, traditional antidepressants including SSRIs demonstrate certain limitations in treatment efficacy (Eke et al., 2016). Therefore, natural products extracted from plants have become attractive sources of alternatives to drugs. Plants, such as saffron and *hypericum perforatum*, have shown potential. The evidence from Tables 2, 3 indicates that plants/herbs have shown both clinical and preclinical efficacy in improving PND.

**Table 2:** Clinical trials of dietary supplements and herbal medicines related to perinatal depression (mainly RCT).

Drug	Scale	Sample	Dose	Therapeutic effect	References
Omega-3 supplementation	EPDS	Sixty pregnant women identified as being at risk for PPD	Fish oil capsules or placebo for 16 weeks	Women in the fish oil group presented a higher reduction on the EPDS score from the second to the third trimester	Vaz et al. (2017)
Zinc and Magnesium Supplements	EPDS, STAI	Zinc sulfate, magnesium sulfate, and placebo groups (*n* = 33 per group)	27-mg zinc sulfate tablet, 320-mg magnesium sulfate tablet or placebo tablet for 8 weeks	No significant difference was observed between groups in mean scores of depressive symptoms, state anxiety, and trait anxiety	Fard et al. (2017)
DHA supplementation	EPDS, BSID	1,197 women with DHA Supplementation and 1,202 women with control Supplementation	Fish oil capsules (providing 800 mg/d of DHA) or matched vegetable oil capsules without DHA from study entry to birth	The percentage of women with high levels of depressive symptoms during the first 6 months postpartum did not differ between the DHA and control groups	Makrides et al. (2010)
Selenium supplements	EPDS	166 primigravid pregnant women in the first trimester of pregnancy	100 μg of selenium vs. placebo per day until delivery	The mean EPDS score in the selenium group was significantly lower than that of the control group (*p* < 0.05)	Mokhber et al. (2011)
Vitamin D and calcium	EPDS	81 women with a PPD score >12 participated	50,000 IU vitamin D3 fortnightly + 500 mg calcium carbonate daily; or vitamin D3 + placebo of calcium carbonate, or placebo of vitamin D3 + placebo of calcium carbonate daily (placebo group) for 8 weeks	The PPD score had more reduction in the vitamin D + calcium and vitamin D + calcium placebo groups than that of the placebo group (*p* < 0.05)	Amini et al. (2022)
Omega-3 fatty acids	EPDS, HDRS	26 women	Fish oil or placebo for 6 weeks	There was no significant difference in depression scores between those receiving fish oil and those receiving the placebo	Rees et al. (2008)
Omega-3 fatty acids	EPDS, HDRS	Subjects were randomized to 0.5 g/day (*n* = 6), 1.4 g/day (*n* = 3), or 2.8 g/day (*n* = 7)	0.5/1.4/2.8 g Omega-3 fatty acids daily, no placebo	Across groups, post-treatment EPDS and HRSD mean scores were 9.3 and 10.0. Percent decreases on the EPDS and HRSD were 51.5 and 48.8%, respectively	Freeman et al. (2006)
*Lactobacillus rhamnosus* HN001	EPDS, STAI	423 women	HN001 at a dose of 6 × 10^9^ colony-forming units (cfu) or placebo until 6 months postpartum if breastfeeding	Women who received HN001 had significantly lower depression and anxiety scores in the postpartum period	Slykerman et al. (2017)
Saffron	HDRS	Receive either saffron (*n* = 34) or fluoxetine (*n* = 34)	A capsule of saffron or a fluoxetine capsule twice daily for 6 weeks	13 (40.60%) patients in the saffron group experienced complete response (≥50% reduction in HDRS score) compared with 16 (50%) in the fluoxetine group and the difference between the 2 groups was not significant	Kashani et al. (2017)
Saffron	BDI-II	Breastfeeding mothers with mild-to-moderate depression (*n* = 60)	15 mg/Bid saffron vs. an equivalent dose of placebo	In the final assessment, 96% of the saffron group were in remission compared to 43% of the placebo group (*p* < 0.01). The complete response rates were 6% for the placebo group and 66% for the saffron group	Tabeshpour et al. (2017)
Crocin	BDI-II, BAI	64 women with mild to moderate PPD	Crocin (15 mg) vs. sertraline (50 mg) daily for 3 months	The mean of the BDI-II score in the crocin group decreased after 3 months from 20.75 to 4.93 (*p* = 0.0001). In the sertraline group, the mean score of BDI-II decreased after 3 months from 21.06 to 2.37 (p = 0.0001). No significant difference was observed between crocin and sertraline after the clinical trial (*p* = 0.5)	Kolahdooz et al. (2023)
Lavender Tea	PSQS, EPDS	80 Taiwanese postnatal women with poor sleep quality	One cup of lavender tea after spending time to appreciate and smell the aroma each day for a period of 2 weeks vs. regular postpartum care only	Experimental group participants perceived less fatigue and depression and showed greater bonding with their infant compared with the control group. However, the scores for all four instruments were similar for both groups at the 4-week posttest	Chen and Chen (2015)
Chamomile tea	PSQS, EPDS	80 Taiwanese postnatal women with poor sleep quality	Chamomile tea for a period of 2 weeks vs. regular postpartum care only	Compared with the control group, the experimental group demonstrated significantly lower scores of physical-symptoms-related sleep inefficiency and the symptoms of depression. However, the scores for all three instruments were similar for both groups at 4-week post-test	Chang and Chen (2016)

RCT, Randomized controlled trial; EPDS, Edinburgh Postnatal Depression Scale; STAI, State–trait anxiety inventory; DHA, Docosahexaenoic acid; BSID, Bayley Scales of Infant development; HDRS, Hamilton Depression Rating Scale; PSQS, Postpartum Sleep Quality Scale; BDI-II, Beck Depression Inventory-Second Edition; BAI, Beck Anxiety Inventory.

**Table 3:** Herbal-based interventions of postpartum depression and their mechanisms of action (focusing on preclinical evidence from the last decade).

Herbal medicine	Mechanisms	Safety evaluation	Major active constituents		Preclinical evidence
Saffron (Iridaceae) Iris L.	Increase the BDNF and VGF Inhibit the uptake of dopamine, norepinephrine, and serotonin Antioxidant, anti-inflammatory Regulate microbiota Hypothalamus-pituitary–adrenal-modulating effects	Mild adverse reactions were shown in clinical trials No evaluation of safety during lactation	Crocin 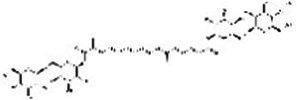	Safranal 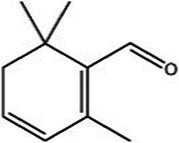	Chen et al. (2023), Pontifex et al. (2022), Monchaux De Oliveira et al. (2021), and Wauquier et al. (2022)
St John’s wort (Guttiferae) Garcinia L.	Modulates monoamine neurotransmitters Glutamate regulation Regulate microbiota Neuroprotective effect Anti-inflammatory and regulate glucocorticoid metabolism Target transient receptor potential canonical 6 channel (TRPC6)	Low breast excretion (Moretti et al., 2009; Zepeda et al., 2022) Induce cytochrome P450 CYP enzyme system and the P-gp drug transporter (Soleymani et al., 2017) Safety Assessment *in vitro* (Spiess et al., 2022)	Hypericin 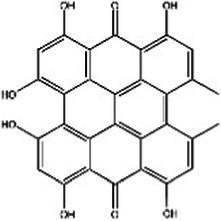	Hyperforins 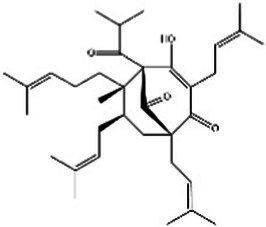	Zhai et al. (2022), Lei et al. (2023) Bonaterra et al. (2017), and El Hamdaoui et al. (2022)
Lavender (Labiatae) Lavandula L.	Inhibits serotonergic target SERT Modulate NMDA receptors Regulate monoaminergic system	No evaluation of safety during lactation Safety Assessment *in vitro* (Dos Reis et al., 2021)	Linalool 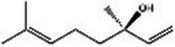	Linalyl acetate 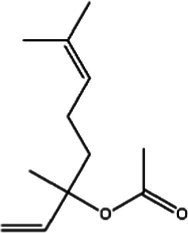	Guzmán-Gutiérrez et al. (2015), Dos Santos et al. (2018), and López et al. (2017)
Chamomile (Compositae) Chrysanthemum L.	Modulate the BDNF Modulate monoamine neurotransmitters	No evaluation of safety during human lactation	Apigenin 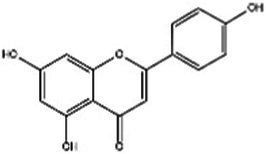	Luteolin 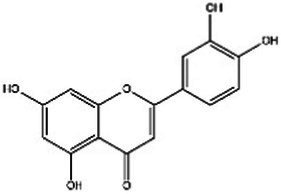	Ahmad et al. (2022)
Basilicum (Labiatae) Ocimum	Increase the BDNF and GR immunoexpression Reduce the corticosterone level	No evaluation of safety during human lactation	Methyl eugenol 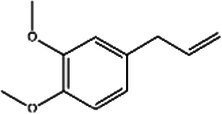	Eugenol 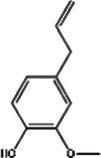	Ali et al. (2017)
Curcuma (Zingiberaceae) Curcuma L.	Modulate monoamine neurotransmitters Antioxidant, anti-inflammatory Neuroprotective effect Kynurenine pathway Modulate NMDA receptors	No significant toxicity in pregnant animals (Soleimani et al., 2018) Mild adverse reactions were shown in clinical trials No evaluation of safety during human lactation	Curcumin 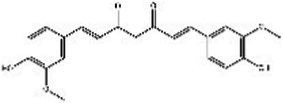		Zhang et al. (2019), Fan et al. (2021), Fan et al. (2018), Zhang et al. (2013), and Jiang et al. (2013)
Rosa (Rosaceae) Rosa L.	Modulate monoamine neurotransmitters	No evaluation of safety during lactation	Linalool 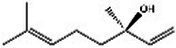	2-phenylethanol 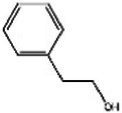	Dos Santos et al. (2018), Ueno et al. (2019), and Matias Nascimento Maia et al. (2021)
Salvia (Labiatae) Salvia L.	Modulates monoamine neurotransmitters Hypothalamus-pituitary–adrenal-modulating effects Antioxidant, anti-inflammatory	No evaluation of safety during lactation	Salvinorin A 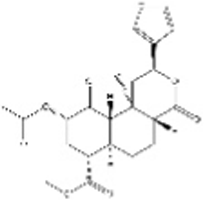	Salvianolic acid B 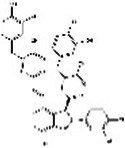	Lee et al. (2014) and Jiang et al. (2017)

All molecular images were obtained from the ChemSpider database. BDNF, Brain-Derived Neurotrophic Factor; VGF, VGF nerve growth factor; NMDA, N-methyl-D-aspartate receptor; GR, Glucocorticoid Receptor.

#### 2.3.1 Saffron

*In vivo* and *in vitro* studies suggest that saffron’s antidepressant effects arise from polypharmacological mechanisms targeting the modulation of the microbiota, neuroinflammation (TNF-α/IL-6 via NF-κB inhibition), monoamine modulation (dopamine, norepinephrine, and serotonin downregulation), HPA axis regulation (cortisol downregulation), and neuroprotection (BDNF upregulation) (Chen et al., 2023; Pontifex et al., 2022; Monchaux De Oliveira et al., 2021; Wauquier et al., 2022). Kashani et al. (2017) evaluated the efficacy of saffron capsules and fluoxetine in the treatment of mild to moderate PND in a randomized controlled trial (RCT). Of the saffron patients, 40.60% (13 cases) had complete responses (50% ≥ HDRS score reduction), compared with 50% (16 cases) in the fluoxetine group. There was no significant difference between the groups in efficacy and adverse reactions (Kashani et al., 2017). Tabeshpour et al. (2017) conducted a randomized placebo-controlled trial on saffron. In the final evaluation, the complete response rate was 6% in the placebo group and 66% in the saffron group. These clinical results support the basic anti-PND studies of saffron, suggesting that saffron may be a safe alternative for improving PND symptoms (Tabeshpour et al., 2017). Despite these promising results, larger trials and further safety evaluations are needed before saffron can be recommended as a first-line treatment for PND.

#### 2.3.2 St. John’s wort

St. John’s wort has been used for a long time to treat everything from anxiety to sleep disorders. Hypericins and hyperforins are the main pharmacodynamic components of St. John’s wort (Table 3). St. John’s wort extract can increase the concentration of dopamine, norepinephrine through multiple pathways and have a broad spectrum antidepressant effect (Linde et al., 2008). Notably, much of its use goes beyond medical guidelines, which also creates potential risks for users. Linde et al. (2005) summarized the double-blind controlled trials on St. John’s wort extract, most using the HAMD score as the efficacy criterion. St. John’s wort was shown to be at least as effective as conventional antidepressants and significantly better than the placebo group. Regarding the significant breastfeeding concern, there is evidence that hypericin from St. John’s wort is excreted into breast milk at low levels (Klier et al., 2006). It is noteworthy that St. John’s Wort serves as a potent inducer of CYP 3A4. Additionally, evidence indicates that it may act as a weak monoamine oxidase inhibitor (MAOI). Consequently, the potential for drug interactions represents a significant safety concern, particularly when co-administered with other SSRIs (e.g., sertraline and Paroxetine), which could elevate the risk of 5-HT Syndrome (Zhou et al., 2004; Sacher et al., 2011). However, although there is partial preclinical evidence and perinatal safety evaluations of St. John’s wort, there is still a lack of reliable large RCT evidence on the safety and efficacy of its use for PND.

#### 2.3.3 Aromatic essential oils (EOs)

Aromatherapy is mainly based on the volatile components of natural products. Plants rich in volatile oils, such as labiaceae and ruticaceae, are often chosen in the treatment of mental disorders (Tsai et al., 2020). Aromatherapy is used globally to alleviate insomnia, depression, anxiety, and certain cognitive disorders. A Taiwanese study assessed the benefits of indoor gardening and inhaling essential oils from *Pseudotsuga menziesii* and *Lavandula angustifolia* for 15 women. Physiological measurements showed decreased sympathetic and increased parasympathetic nerve activity, indicating relaxation. Psychological measurements showed reduced STAI-S anxiety scores after gardening and inhaling the essential oils (Chung et al., 2024). Hachul et al. conducted two placebo-controlled studies on the effects of lavender essential oil on sleep in postmenopausal women, comparing sunflower oil and lavender essential oil. Participants in the lavender group showed improvements in overall sleep patterns, quality, and efficiency, as well as benefits in almost all areas of quality of life (Lucena et al., 2024; Dos Reis et al., 2021). In addition, aromatherapy with lavender essential oil is believed to help manage Premenstrual Syndrome (PMS) by reducing symptoms such as anxiety, depressed mood, nervousness, pain, bloating, and negative thoughts. However, the study lacked a placebo (Uzunçakmak and Ayaz, 2018). In a clinical trial, 140 postpartum women were randomly assigned to either an aromatherapy group or a non-aromatherapy group (Kianpour et al., 2016). The aromatherapy intervention involved inhaling 3 drops of lavender essential oil every 8 h for 4 weeks, while the control group received usual care after hospital discharge. Results showed that stress, anxiety, and depression scores were significantly lower in the aromatherapy group compared to the control group.

Although essential oils have shown some advantages in improving depression and relieving anxiety, the current view that aromatherapy improves or treats depression is still controversial (Jafari-Koulaee et al., 2020; Chen et al., 2022). On the one hand, in the absence of sufficiently reliable medical evidence, placebo-controlled trials on aromatherapy are difficult to design because aromatherapy focuses on smell, and the subject can judge the group by the smell. Additionally, the use of essential oils carries potential risks, including allergic reactions, skin irritation, and toxicity if not properly managed. Further research is required to clarify the therapeutic potential and safety of aromatherapy for PND.

### 2.4 Dietary habit and dietary supplements

For the treatment of PND through natural intervention strategies, some research has focused on the use of dietary supplements to prevent prodromal symptoms. There is evidence that deficiencies in omega-3 polyunsaturated fatty acids (PUFAs), vitamin D, and other key elements, such as iron, zinc, magnesium, and selenium, are highly associatead with PND symptoms (Rupanagunta et al., 2023). Herring fish oil supplements reduced hippocampal corticosterone concentration and plasma corticosterone levels, as well as proinflammatory cytokines (IL-1β, INF-γ and TNF-α) and improved depressive symptoms in model animals with PND (Arbabi et al., 2014). However, in several randomized placebo-controlled trials involving omega-3 in the prevention of PND, researchers found no significant benefit of PUFAs rich fish oil supplements compared with a placebo in the prevention of PND (Freeman et al., 2006; Rees et al., 2008; Vaz et al., 2017; Hulkkonen et al., 2021; Makrides et al., 2010). Amini et al. (2022) conducted the first clinical trial of the effect of vitamin D and calcium supplements on women with PND symptoms. The results showed that vitamin D supplements reduced PND scores; in addition, women taking vitamin D alone lowered their PND scores more than those taking vitamin D + Ca or the placebo (Amini et al., 2022). In another study of zinc and magnesium supplements, changes in depressive symptoms were not satisfactory (Fard et al., 2017).

Emerging evidence also highlights the potential of probiotics as adjunctive interventions for PND. A randomized, double-blind, placebo-controlled trial (*n* = 423) demonstrated that maternal supplementation with *Lactobacillus rhamnosus* HN001 during pregnancy and postpartum (until 6 months breastfeeding) significantly reduced postpartum depression (mean EPDS 7.7 vs. 9.0, *p* = 0.037) and anxiety scores (12.0 vs. 13.0, *p* = 0.014) compared to placebo, with a 56% lower risk of clinical anxiety (OR = 0.44, *p* = 0.002) (Slykerman et al., 2017). However, these effects were observed as secondary outcomes in a trial primarily designed to assess infant eczema, underscoring the need for dedicated trials. Another study in overweight women (*n* = 439) found no synergistic benefits of combining probiotics with fish oil for mood symptoms, though baseline dietary quality inversely correlated with depressive/anxiety trajectories (Hulkkonen et al., 2021). Despite promising safety profiles (breastfeeding-compatible), mechanistic insights into gut-brain axis modulation remain limited.

Despite their low cost and minimal side effects, dietary supplements cannot currently be recommended as a standalone treatment for PND due to insufficient evidence of their efficacy. More rigorous clinical trials are needed to confirm their effectiveness and establish appropriate dosages for perinatal populations.

## 3 Conclusion

Perinatal depression (PND), a multifactorial disorder involving neuroendocrine dysregulation and gut-brain axis disruption, demands innovative therapeutic strategies. This review highlights the potential of alternative interventions—such as saffron, St. John’s wort, and lavender aromatherapy—to alleviate symptoms, with saffron showing efficacy comparable to SSRIs in short-term trials. However, critical limitations persist: insufficient evidence on lactation safety (e.g., transfer of bioactive compounds into breast milk), variability in herbal formulations, and a lack of long-term outcome data. Dietary supplements like omega-3s and vitamin D yield inconsistent clinical results, underscoring the need for standardized dosing and robust trial designs. While these therapies offer safer perceptions for patients, their clinical application requires rigorous risk–benefit evaluation, particularly for breastfeeding mothers. Future research must prioritize large-scale RCTs, mechanistic studies, and safety monitoring to bridge the gap between preclinical promise and clinical utility. By integrating emerging evidence with practical guidelines, this work aims to support informed decision-making for PND management, balancing maternal mental health with infant safety.

## 4 Discussion

PND receives varying levels of attention in different countries/regions. Fisher et al. (2012) concluded that women in middle-and low-income countries had a higher incidence of perinatal mental disorders. A wealth of high-quality epidemiological, clinical, and healthcare-related evidence surrounding PND has been published from high-income/developed countries. In a study of 112 middle-and low-income countries, 80–90% of these least developed countries lacked local evidence on perinatal mental disorders (Fisher et al., 2012). Moreover, women from the most economically and socially vulnerable groups have the highest incidence rates. Because of constraints, such as the lack of access to healthcare (related to economic status), certain cultural and social factors (including discrimination against women, sex biases toward future generations, traditional cultural ostracism of psychiatric medicine, etc.), the legitimate needs of these women facing the risks of PND are not only ignored and neglected but their fundamental rights to perinatal preventive care are also violated (Fisher et al., 2010). Further research and action are needed to address the differences between countries and social groups in diagnosing and treating PND. This includes discussing potential interventions and policies to improve the identification and management of PND among low-income countries and marginalized populations, and focusing on cultural and social factors that can lead to shame and discrimination against women with perinatal depression. Cross-cultural and interdisciplinary cooperation on a global scale is needed to understand the etiology, prevention, and treatment of perinatal depression in different cultural backgrounds, and to provide better PND diagnosis and treatment for women all over the world.

Growing preclinical and clinical evidence suggests that non-traditional treatment modalities, such as the ones described in this review, are gaining increasing attention in the field of perinatal depression. These treatments include a wide range of interventions, such as herbal medicine, dietary supplements, and aromatherapy, all of which have shown some promise in alleviating symptoms associated with perinatal depression. However, current data are insufficient to recommend these as monotherapies for perinatal depression, and several issues cannot be ignored. First, the quality of these clinical trial data must be considered (including experimental design, dosing plan and intervention measures). In Table 2, two clinical trials of omega-3 involved fewer than 30 participants (Freeman et al., 2006; Rees et al., 2008). Regarding control group interventions, three clinical trials in Table 2 lacked placebo controls, and two were positive controls using fluoxetine or sertraline. The lack of a placebo control group similarly raises concerns about the quality of these clinical trials (Baldwin et al., 2003). Furthermore, the quality of trial design often lacks rigor. For instance, blinding is difficult to achieve in studies involving aromatherapy, leading to potential bias from both participants and researchers. The heterogeneity of interventions, including variations in the type, dosage, frequency, and duration of herbal and dietary supplements, complicates the comparison and evaluation of these studies. Heterogeneity is another concern, as omega-3 supplements in Table 2 come in many forms, with different omega-3 dosages and different proportions of EPA and DHA in different supplements, making it challenging to evaluate these studies.

The challenge with using herbal medicine in (PND) lies in the lack of human data. In Table 3, nine types of Chinese herbal medicines are listed with preclinical evidence for PND treatment, but only 2–3 of these herbs have clinical trial data involving women in the perinatal period in Table 2. For example, among the Chinese herbs mentioned in this article, saffron is more commonly used in women and has rarely been studied as a monotherapy for postpartum depression (Tabeshpour et al., 2017; Kashani et al., 2017). On the one hand, the metabolic pathways of drugs in animals and humans can differ significantly, and animal models often only partially mimic the pathological features of human diseases, failing to fully reproduce their complexity and diversity. This is especially true for psychiatric disorders, such as depression, where animal models can only simulate certain symptoms or pathological mechanisms. Common animal models for postpartum depression, such as hormone resistance models, chronic unpredictable stress models, and cortisol induction models, lack complexity and focus on single factors, which is a significant limitation. Therefore, clinical efficacy in humans cannot always be inferred from *in vivo* and *in vitro* pharmacodynamic evidence. Despite these limitations, such information does provide potential guidance for future research and helps in understanding the underlying mechanisms and potential therapeutic targets. Breastfeeding significantly influences researchers’ approach to using herbal medicine in perinatal mothers. There is a prevailing public belief that natural products are generally safe for pregnant women and fetuses; however, this assumption is often incorrect. A survey showed that nearly 40% of pregnant women use of the medicinal plants may not be safe (Bernstein et al., 2021). The use of St. John’s wort does not impact breastfeeding, but it may act as a CYP 3A4 inducer. Therefore, there is a potential risk for interaction with other herbal or traditional drugs during pregnancy (Dugoua et al., 2006; Markowitz and DeVane, 2001). The dilemma is the lack of studies or only partial studies on the efficacy and safety of most conventional drugs during pregnancy. Therefore, the view that “natural is harmless” needs to be cautioned against, and caution should always be exercised until sufficient reliable studies of perinatal use of herbal medicines are available. In addition to this, although omega-3 fatty acids have shown benefits in treating major depression, a significant number of studies have not supported their effectiveness in preventing postpartum depression. Similarly, other dietary supplements have also failed to demonstrate a clear preventive role in this context (Miller et al., 2013).

Although randomized controlled trials are regarded as the gold standard for evaluating monotherapies, this does not diminish the potential value of alternative therapies when used in combination. Herbal medicine often exerts a synergistic anti-depressant effect through the combination of various herbs and their ingredients. For example, the combination of bupleurum and peony can enhance the anti-depressant effect (Li et al., 2021). Herbal prescriptions like Xiaoyao powder and are commonly used for this purpose (Gao et al., 2022; Wang et al., 2023). Additionally, combining these herbal treatments with psychotherapy can further enhance their effectiveness. Medication combined with psychotherapy can enhance the treatment of specific symptoms of depression and anxiety (Bozzatello et al., 2020).

Compared with traditional antidepressants (including monoamine oxidase inhibitors (MAOs) and SSRIs), there is evidence that perinatal mothers are more likely to accept herbs or dietary supplements that appear to be safer, healthier, easier and cheaper. However, from our research, their effectiveness and safety data are not as strong as the public thinks. Another problem is that when consumers choose what they think is a safe treatment, many applications are actually outside the professional guidance of the clinician, which can create additional risks. Therefore, taking into account the fact that perinatal mothers prefer perinatal medications and alternative therapies, their knowledge of the effectiveness and potential risks of these interventions should be increased.
